# *N*′-(1,3-Benzo­thia­zol-2-yl)benzene­sulfono­hydrazide: crystal structure, Hirshfeld surface analysis and computational chemistry

**DOI:** 10.1107/S2056989019003980

**Published:** 2019-03-29

**Authors:** Thomas C. Baddeley, Marcus V. N. de Souza, James L. Wardell, Mukesh M. Jotani, Edward R. T. Tiekink

**Affiliations:** a Department of Chemistry, University of Aberdeen, Meston Walk, Old Aberdeen AB24 3UE, Scotland; b Instituto de Tecnologia em Fármacos e Farmanguinhos, Fundação Oswaldo Cruz, 21041-250 Rio de Janeiro, RJ, Brazil; c Instituto de Tecnologia em Fármacos Farmanguinhos, Fundação Oswaldo Cruz, 21041-250 Rio de Janeiro, RJ, Brazil; d Department of Physics, Bhavan’s Sheth R. A. College of Science, Ahmedabad, Gujarat 380001, India; e Research Centre for Crystalline Materials, School of Science and Technology, Sunway University, 47500 Bandar Sunway, Selangor Darul Ehsan, Malaysia

**Keywords:** crystal structure, benzo­thia­zole, sulfonyl­hydrazin­yl, hydrogen bonding, Hirshfeld surface analysis, computational chemistry

## Abstract

The asymmetric unit of the title compound, C_13_H_11_N_3_O_2_S_2_, comprises two independent mol­ecules (*A* and *B*); the crystal structure was determined by employing synchrotron radiation. The mol­ecules exhibit essentially the same features with an almost planar benzo­thia­zole ring (r.m.s. deviation = 0.026 and 0.009 Å for *A* and *B*, respectively), which forms an inclined dihedral angle with the phenyl ring [28.3 (3) and 29.1 (3)°, respectively]. A difference between the mol­ecules is noted in a twist about the N—S bonds [the C—S—N—N torsion angles = −56.2 (5) and −68.8 (5)°, respectively], which leads to a minor difference in orientation of the phenyl rings. In the mol­ecular packing, *A* and *B* are linked into a supra­molecular dimer *via* pairwise hydrazinyl-N—H⋯N(thiazol­yl) hydrogen bonds. Hydrazinyl-N—H⋯O(sulfon­yl) hydrogen bonds between *A* mol­ecules assemble the dimers into chains along the *a*-axis direction, while links between centrosymmetrically related *B* mol­ecules, leading to eight-membered {⋯HNSO}_2_ synthons, link the mol­ecules along [001]. The result is an undulating supra­molecular layer. Layers stack along the *b*-axis direction with benzo­thia­zole-C—H⋯O(sulfon­yl) points of contact being evident. The analyses of the calculated Hirshfeld surfaces confirm the relevance of the above inter­molecular inter­actions, but also serve to further differentiate the weaker inter­molecular inter­actions formed by the independent mol­ecules, such as π–π inter­actions. This is also highlighted in distinctive energy frameworks calculated for the individual mol­ecules.

## Abstract 2

Two conformationally similar mol­ecules comprise the asymmetric unit of the title compound. In the crystal, hydrazinyl-N—H⋯N(thia­zol­yl) and hydrazinyl-N—H⋯O(sulfon­yl) hydrogen bonds assemble the mol­ecules into an undulating supra­molecular layer parallel to (010).

## Chemical context   

Benzo­thia­zole derivatives have attracted attention over a long period of time because of their wide spectrum of biological activities and the benzo­thia­zole framework remains today an important scaffold for the design and synthesis of active mol­ecules (Gill *et al.*, 2015 ▸; Reshma *et al.*, 2017 ▸; Thakkar *et al.*, 2017 ▸; Dar *et al.*, 2016 ▸). Among recent reports on benzo­thia­zole derivatives are those on 2-aryl­idenehydrazinylbenzo­thia­zoles, which include anti-tumour activities (Lindgren *et al.*, 2014 ▸; Nogueira *et al.*, 2010 ▸; Katava *et al.*, 2017 ▸) and anti-tuberculosis activity against *M. tuberculosis* ATTC 27294 (Pinheiro *et al.*, 2019 ▸); crystal structure determinations have also been included in each of these studies. Less work has been carried out on other 2-hydrazinylbenzo­thia­zoles, such as the arenesulfonyl derivatives, 2-(2-Ar-sulfonyl­hydrazin­yl)-1,3-benzo­thia­zoles. Only a brief report has appeared on their anti-microbial activities (Rao *et al.*, 2004 ▸) and only very recently has a crystal structure determination of the species where Ar = 3-O_2_NC_6_H_4_ has been described (Morscher *et al.*, 2018 ▸). Herein, as a continuation of the latter studies, the crystal and mol­ecular structures of the title compound, (I), are described. The X-ray intensity data were collected on a small sample with synchrotron radiation and crystallography revealed the presence of two independent mol­ecules in the asymmetric unit. In order to ascertain the individual contributions of these mol­ecules to the mol­ecular packing, an analysis of the calculated Hirshfeld surfaces was also conducted.
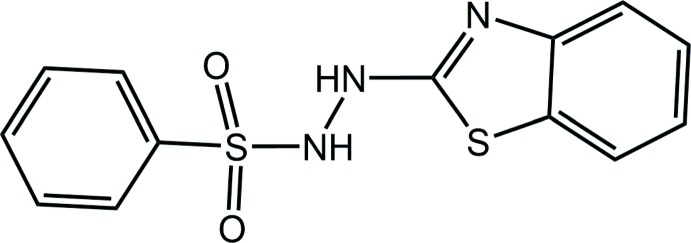


## Structural commentary   

**Figure 1:**
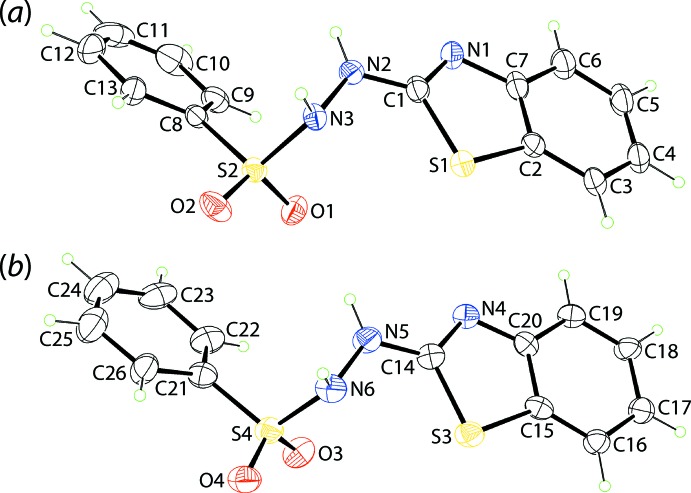
The mol­ecular structures of the two independent mol­ecules of (I), showing the atom-labelling scheme and displacement ellipsoids at the 25% probability level.

**Figure 2:**
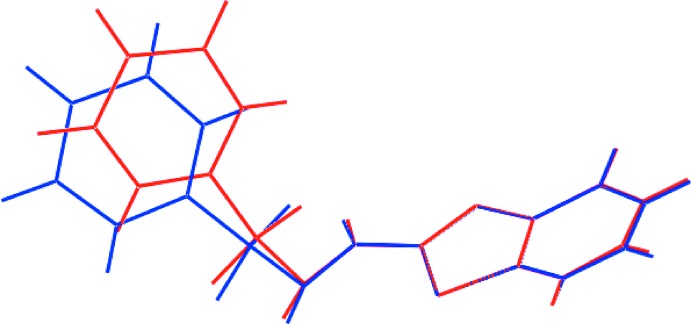
An overlay diagram of the S1-containing (red image) and S3-containing (blue) mol­ecules. The mol­ecules have been overlapped so the thia­zole rings are coincident.

Two independent mol­ecules comprise the asymmetric unit of (I) and their mol­ecular structures are shown in Fig. 1 ▸. In the S1-containing mol­ecule, the r.m.s. deviation of the nine atoms forming the benzo­thia­zole ring is 0.026 Å with maximum deviations out of the plane being 0.038 (8) Å for the C4 atom and 0.029 (6) Å for C2. The equivalent values for the S3-mol­ecule are 0.009 Å with deviations of 0.010 (6) Å for the C16 atom and 0.013 (7) Å for C15. The dihedral angle between the benzo­thia­zole and phenyl rings is 28.3 (3) and 29.1 (3)° for the S1- and S3-mol­ecules, respectively, indicating very similar overall conformations for the mol­ecules. This is reflected in the small r.m.s. bond and angle fits of 0.0196 Å and 1.126°, respectively (Spek, 2009 ▸). However, as seen from Fig. 2 ▸, the twist in the mol­ecules about the N—S bonds differs, as seen in the disparity of about 12° in the C8—S2—N3—N2 [−56.2 (5)°] and C21—S4—N6—N5 [−68.8 (5)°] torsion angles. This leads to a lateral mismatch in the phenyl groups.

## Supra­molecular features   

**Figure 3:**
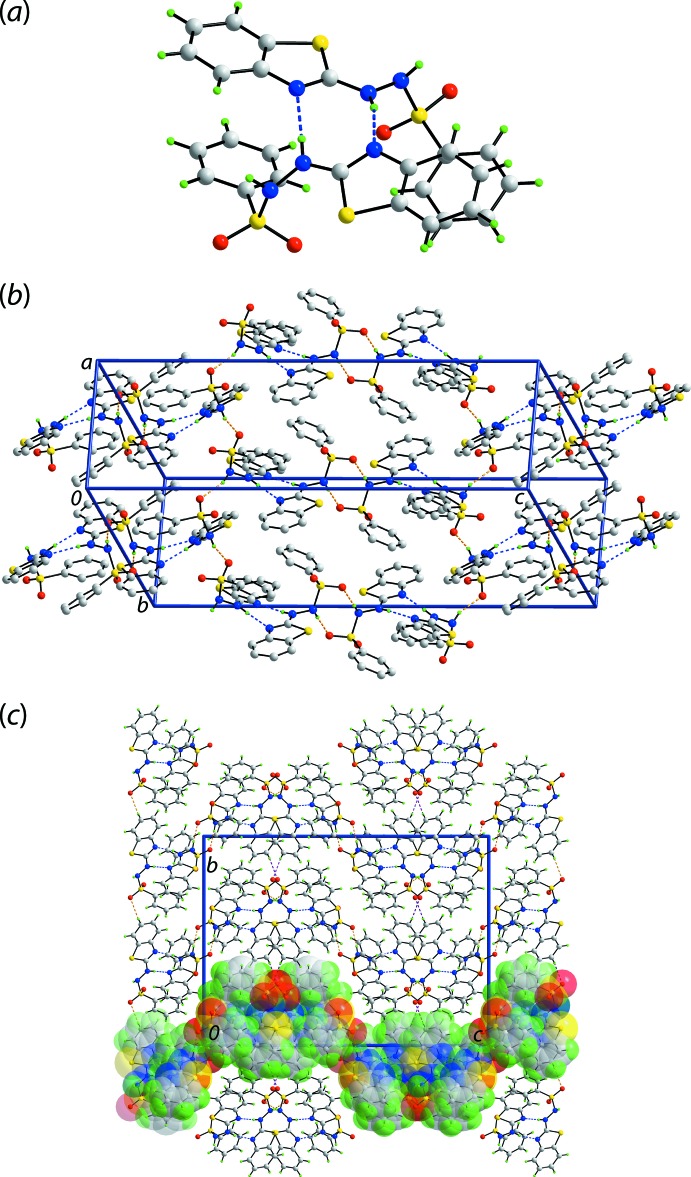
Supra­molecular association in the crystal of (I): (*a*) dimeric aggregate sustained by hydrazide-N—H⋯N(thia­zol­yl) hydrogen bonds shown as blue dashed lines, (*b*) supra­molecular layer in the *ac* plane whereby the dimers of (*a*) are linked by hydrazide-N—H⋯O(sulfon­yl) hydrogen bonds (orange dashed) lines (non-acidic hydrogen atoms have been omitted) and (*c*) a view of the unit-cell contents shown in projection down the *a* axis. The benzo­thia­zole-C—H⋯O(sulfon­yl) inter­actions are shown as purple dashed lines and one layer has been highlighted in space-filling mode.

The mol­ecular packing of (I) features hydrazinyl-N—H⋯N(thia­zol­yl) and hydrazinyl-N—H⋯O(sulfon­yl) con­ven­tional hydrogen bonds, Table 1 ▸. The hydrazinyl-N—H⋯N(thia­zol­yl) hydrogen bonds serve to link the two mol­ecules comprising the asymmetric unit into a dimeric aggregate *via* an eight-membered {⋯HNCN}_2_ synthon, Fig. 3 ▸(*a*). Each of the remaining hydrazinyl-N—H atoms forms a hydrogen bond to a sulfonyl-O atom derived from a symmetry-related mol­ecule. The hydrazinyl-N—H⋯O(sulfon­yl) hydrogen bonds involving S1-mol­ecules give rise to *C*(4), {⋯HNSO}_*n*_, supra­molecular chains along the *a*-axis direction. By contrast, those involving the S3-mol­ecules occur between centrosymmetrically related mol­ecules and lead to an eight-membered {⋯HNSO}_2_ synthon. The latter serve to link mol­ecules along the *c-*axis direction so that a supra­molecular layer, with an undulating topology, in the *ac* plane results, Fig. 3 ▸(*b*). The distinctive modes of the hydrazinyl-N—H⋯O(sulfon­yl) hydrogen bonds just outlined provide a clear differentiation between the mol­ecules. The most obvious points of contact to link layers along the *b*-axis direction are of the type benzo­thia­zole-C—H⋯O(sulfon­yl), Table 1 ▸ and Fig. 3 ▸(*c*).

## Hirshfeld surface analysis   

The Hirshfeld surfaces calculated for (I) were performed following procedures outlined recently (Tan *et al.*, 2019 ▸) and provide additional information on the distinctive contributions made to the mol­ecular packing by the independent mol­ecules.

**Figure 4:**
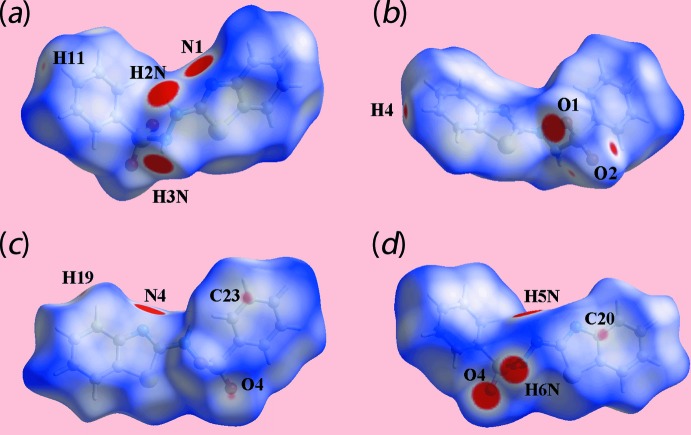
Views of the Hirshfeld surface for (I) mapped over *d*_norm_ for (*a*) and (*b*) the S1-containing mol­ecule (range: − 0.120 to +1.433 arbitrary units) and (*c*) and (*d*) the S3-containing mol­ecule (−0.120 to +1.392 arbitrary units).

On the Hirshfeld surfaces mapped over *d*_norm_ for the S1-containing mol­ecule, Fig. 4 ▸(*a*),(*b*), and the S3-mol­ecule, Fig. 4 ▸(*c*),(*d*), the influence of the hydrazinyl-N—H⋯N(thia­zol­yl) hydrogen bonds sustaining the dimeric aggregate, Table 1 ▸, are evident as broad and bright-red spots near the participating atoms. The presence of inter­molecular N—H⋯O hydrogen bonds involving the hydrazinyl-N3, N6 and sulfonyl-O1, O4 atoms are also viewed as broad and bright-red spots near the respective atoms in the images of Fig. 4 ▸. In addition, the weak C—H⋯O contacts are characterized by the diminutive red spots near the benzo­thia­zolyl-H4 and sulfonyl-O2 atoms, Fig. 4 ▸(*b*), and the faint-red spots near the benzene-H11, benzo­thia­zolyl-H19 and sulfonyl-O2,O4 atoms in Fig. 4 ▸(*a*)–(*c*). The presence of a short inter­atomic C⋯C contact involving atoms C20 and C23 of the S3-mol­ecule, Table 2 ▸, describing π–π stacking inter­actions between symmetry-related S1-thia­zole and benzene (C21–C26) rings is evident as the faint-red spots near these atoms in Fig. 4 ▸(*c*),(*d*).

**Figure 5:**
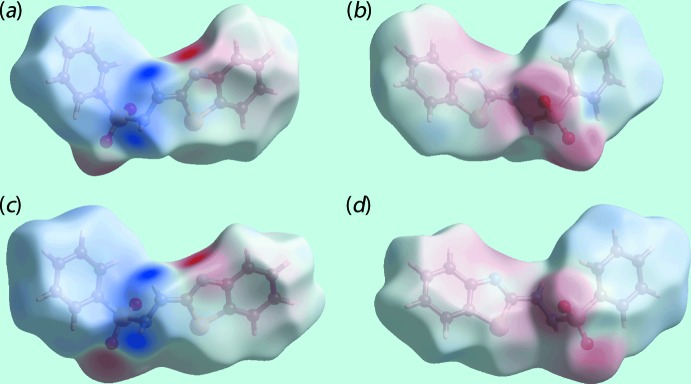
Views of the Hirshfeld surface for (I) mapped over the electrostatic potential for (*a*) and (*b*) the S1-containing mol­ecule (range: −0.137 to +0.175 atomic units) and (*c*) and (*d*) the S3-containing mol­ecule (−0.141 to +0.152 atomic units). The red and blue regions represent negative and positive electrostatic potentials, respectively.

The donors and acceptors of the N—H⋯N and N—H⋯O hydrogen bonds are also viewed as the intense-blue and -red regions corresponding to positive and negative electrostatic potentials on the Hirshfeld surfaces mapped over the calculated electrostatic potentials for the S1- and S3-mol­ecules in the images of Fig. 5 ▸.

**Figure 6:**
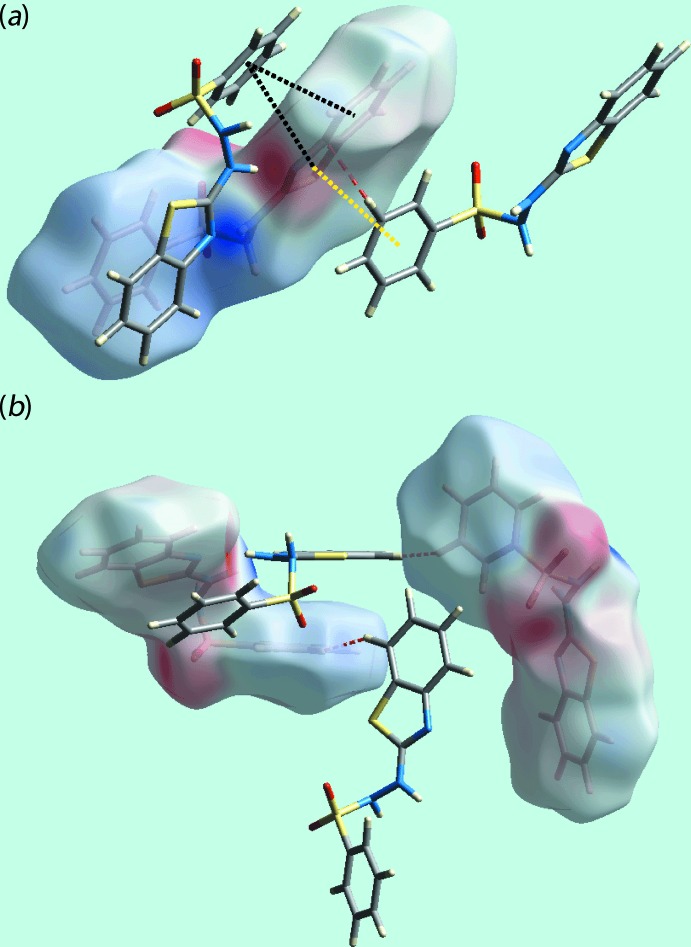
Views of Hirshfeld surfaces mapped over the electrostatic potential highlighting (*a*) π–π stacking between the mol­ecules comprising the asymmetric unit (through black dotted lines) and between symmetry-related mol­ecules (yellow) and short inter­atomic C⋯C contacts (red) and (*b*) short inter­atomic H⋯H contacts through red dashed lines.

An additional distinction in the mol­ecular environments about the crystallographically independent mol­ecules, over and above the hydrazinyl-N—H⋯O(sulfon­yl) hydrogen bonds discussed above, is apparent in terms of their participation in π–π inter­actions, Table 3 ▸. Thus, the benzene and benzo­thia­zole rings of the S3-mol­ecule participate in such contacts in contrast to the involvement of only the benzene ring of the S1-mol­ecule, as illustrated in Fig. 6 ▸(*a*). The influence of the short inter­atomic H⋯H contact between benzene-H10 (S1-mol­ecule) and benzo­thia­zolyl-H16 (S3-mol­ecule) atoms is also illustrated in Fig. 6 ▸(*b*) through the red dashed lines superimposed on Hirshfeld surface mapped over the electrostatic potential.

**Figure 7:**
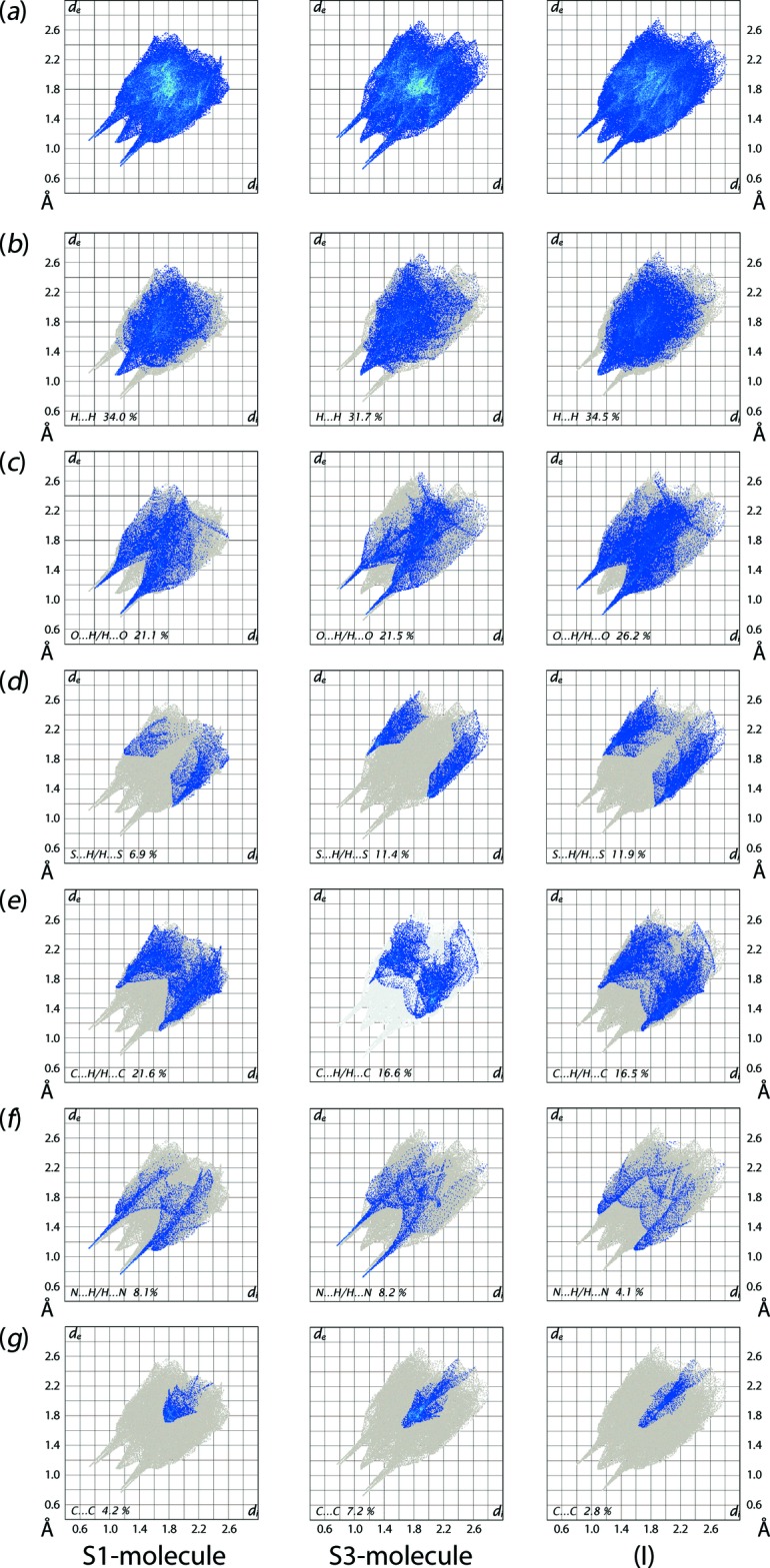
(*a*) The full two-dimensional fingerprint plot for the S1-mol­ecule in (I), the S3-containing mol­ecule and overall (I) and (*b*)–(*f*) those delineated into H⋯H, O⋯H/H⋯O, N⋯H/H⋯N, C⋯H/H⋯C, S⋯H/H⋯S and C⋯C contacts, respectively.

The overall two-dimensional fingerprint plot for the individual S1-and S3-mol­ecules, and entire (I) are shown in Fig. 7 ▸(*a*), and those delineated into H⋯H, O⋯H/H⋯O, S⋯H/H⋯S, C⋯H/H⋯C, N⋯H/H⋯N and C⋯C contacts are illustrated in Fig. 7 ▸(*b*)–(*g*); the percentage contributions from different inter­atomic contacts to their respective Hirshfeld surfaces are qu­anti­tatively summarized in Table 4 ▸. Although the overall fingerprint plots for the S1- and S3-mol­ecules in Fig. 7 ▸(*a*) are only slightly different, their delin­eated fingerprint plots in Fig. 7 ▸(*b*)–(*g*) clearly indicate their distinct modes of supra­molecular association in the crystal.

The fingerprint plots delineated into H⋯H contacts for the S1- and S3-mol­ecules in Fig. 7 ▸(*b*) represent the complementary pair of knife-edge tips at *d*_e_ + *d*_i_ ∼2.2 Å which merge to form the conical tip in the respective plot for overall (I). The pair of spikes at *d*_e_ + *d*_i_ ∼2.0 Å in the fingerprint plot delin­eated into O⋯H/H⋯O contacts for both independent mol­ecules in Fig. 7 ▸(*c*), with nearly the same percentage contributions to the Hirshfeld surfaces (Table 4 ▸), arises owing to the involvement of the atoms of the respective mol­ecules in the inter­molecular N—H⋯O hydrogen bonds which finally superimpose in the plot for overall (I). In the fingerprint plot delineated into N⋯H/H⋯N contacts in Fig. 7 ▸(*f*), the pair of spikes at *d*_e_ + *d*_i_ ∼1.8 Å and 1.9 Å for the S1- and S3-mol­ecules, respectively, represent the presence of the N—H⋯N hydrogen bonds between them, to form the dimeric aggregate shown in Fig. 2 ▸(*a*). These features of the fingerprint plots disappear in the corresponding plot for overall (I) correlating with the decreased the percentage contribution from these contacts to the overall Hirshfeld surface (Table 4 ▸).

The presence of the short inter­atomic C⋯H contact between the atoms of S1-mol­ecules result in the pair of peaks at *d*_e_ + *d*_i_ ∼2.8 Å in the fingerprint plot delineated into C⋯H/H⋯C contacts in Fig. 7 ▸(*e*) for the S1-mol­ecule and for overall (I). The fingerprint plots delineated into S⋯H/H⋯S contacts in the three images of Fig. 7 ▸(*d*) indicate the inter­atomic separations are greater than the sum of the van der Waals radii suggesting their limited influence on the mol­ecular packing. The distinct, arrow-shaped distribution of points with different percentage contributions due to C⋯C contacts illustrated in Fig. 7 ▸(*g*) are due from the different π–π contacts made by the S1- and S3-mol­ecules. The small contributions from the other inter­atomic contacts have negligible effects upon the mol­ecular packing.

## Computational chemistry   

**Figure 8:**
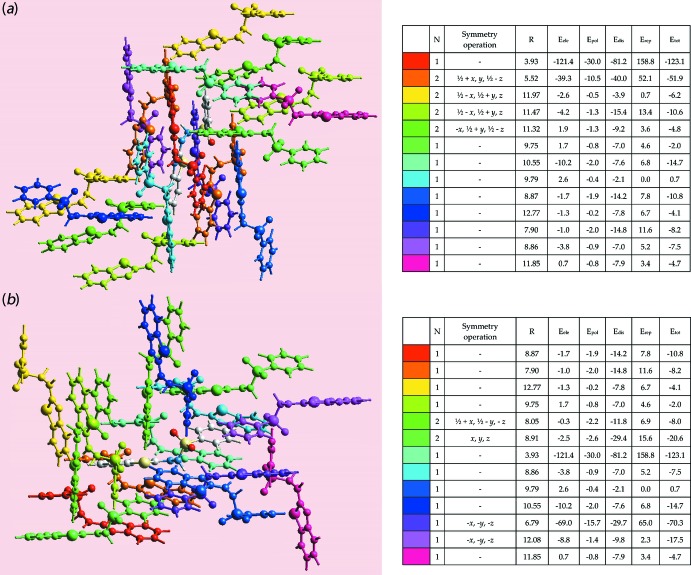
The colour-coded inter­action mapping for the clusters within 3.8 Å of the (*a*) S1-mol­ecule and (*b*) S3-mol­ecule.

The pairwise inter­action energies between the mol­ecules in the crystal are calculated by summing up four energy components, comprising electrostatic (*E*_ele_), polarization (*E*_pol_), dispersion (*E*_dis_) and exchange-repulsion (*E*_rep_) (Turner *et al.*, 2017 ▸). The energies were obtained by using the wave function calculated at the B3LYP/6-31G(*d*,*p*) level of theory for each independent mol­ecule. The individual energy components as well as total inter­action energies relative to the respective reference mol­ecule within the mol­ecular cluster are illustrated in Fig. 8 ▸.

The strength and the nature of the inter­molecular inter­actions in terms of their energies are qu­anti­tatively summarized in Table 5 ▸. The results reveal electrostatic inter­actions to be significant in the N—H⋯N hydrogen bonds which link the two independent mol­ecules in the crystal *via* the {⋯HNCN}_2_ synthon. In the N—H⋯O hydrogen bond involving the S1-mol­ecule, the electrostatic as well as dispersive components are dominant in contrast to a major contribution from only the electrostatic energy for the analogous hydrogen bond formed by the S3-mol­ecule. This result is correlated with the latter hydrogen bonding linking S3-mol­ecules *via* a {⋯HNSO}_2_ synthon as opposed to the chain sustained by the former. The weak inter­molecular C—H⋯O inter­actions in the crystal have major contributions from dispersion energy components. It is also evident from the comparison of the total energies of the inter­molecular inter­actions in Table 5 ▸ that the N—H⋯N hydrogen bonds between the mol­ecules comprising the asymmetric unit are stronger than the N—H⋯O hydrogen bonds, and that the C—H⋯O contacts are significantly weaker than these.

**Figure 9:**
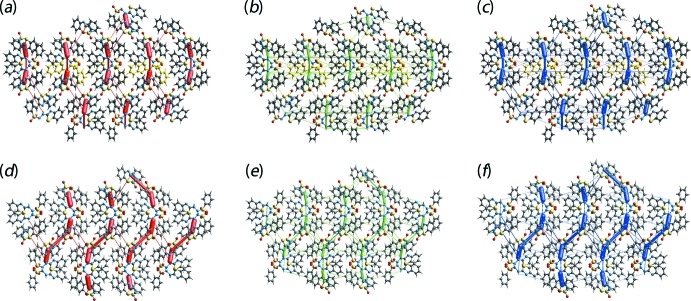
A comparison of the energy frameworks composed of (*a*) electrostatic potential force, (*b*) dispersion force and (*c*) total energy for for the S1-mol­ecule and and (*d*)–(*f*) comparable frameworks for the S3-mol­ecule. The energy frameworks were adjusted to the same scale factor of 50 with a cut-off value of 5 kJ mol^−1^ within 2 × 2 × 2 unit cells.

The magnitudes of the inter­molecular energies are represented graphically in the energy frameworks in Fig. 9 ▸. Here, the supra­molecular architecture of the crystal is viewed through cylinders joining centroids of mol­ecular pairs using red, green and blue colour codes for the energy components *E*_ele_, *E*_disp_ and *E*_tot_, respectively. The radius of the cylinder is proportional to the magnitude of the inter­action energy which have been adjusted to the same scale factor within 2 × 2 × 2 unit cells. The illustrated energy frameworks constructed for clusters of both the independent mol­ecules also indicate their participation in distinct modes of supra­molecular association.

## Database survey   

As indicated in the *Chemical context*, the structure determin­ation of (I) is only the second such analysis for 2-(2-Ar-sulfonyl­hydrazin­yl)-1,3-benzo­thia­zole mol­ecules, the first being the example where Ar = 3-O_2_NC_6_H_4_ (Morscher *et al.*, 2018 ▸); in (I), Ar = C_6_H_5_. In the literature precedent, there are also two independent, but conformationally similar mol­ecules in the asymmetric unit and these, too, are linked into supra­molecular dimers *via* hydrazinyl-N—H⋯N(thia­zol­yl) hydrogen bonds. As reported for the literature structure, the atoms equivalent to N2 and N5 in (I) have significant *sp* ^2^ character based on the sums of the angles about these atoms. This is also true in (I) where the angles sum to 360.2 and 359.2°, respectively. The same considerations led the authors to conclude that the N3 and N6 atoms have some *sp* ^3^ character. Substanti­ating this conclusion, in (I) the sum of the angles amount to 344.0 and 346.4°, respectively. Finally, the C1—N2 and C14—N5 bond lengths of 1.334 (7) and 1.365 (8) Å, respectively, are indicative of some double-bond character, an observation again consistent with the literature precedent.

## Synthesis and crystallization   

The melting point was determined on a Griffin melting point apparatus and is uncorrected. Infrared spectra, as neat powders, were recorded using a Perkin Elmer UATR two instrument, with an ATR Diamond Cell NMR spectra were recorded on a Bruker Avance 400 spectrometer in DMSO-*d*_6_ solution at room temperature. Accurate mass measurements were determined using a Water Mass Spectrometer Model Xevo G2 QT instrument.

Preparation: A solution of 2-hydrazinyl-1,3-benzo­thia­zole (1.66 g, 1 mmol) and benzene­sulfonyl chloride (1.77 g, 1 mmol) in EtOAc (20 ml) was refluxed for 1 h. The reaction mixture was washed with water, the organic layer was collected, dried over magnesium sulfate and rotary evaporated. The residue was recrystallized from an ethanol solution. Yield 82%. The sample used in the structure determination was obtained by slow evaporation of an ethanol solution at room temperature after two days; m.p. 643–465 K. IR (cm^−1^): 3202, 3100–2600 (*br*), 1615, 1583, 1466, 1448, 1325, 1274 1161, 1087, 888, 746, 637. ^1^H NMR (400 MHz, DMSO-*d*_6_): δ 7.15(1H, *t*), 7.31(1H, *br*), 7.40(1H, *br. s*), 7.67(2H, *t*), 7.78(2H, *m*), 7.94(2H, *d*); NH not observed. ^13^C{^1^H} NMR (100 MHz, DMSO-*d*_6_): δ 121.91, 122.22, 125.96, 126.34, 128.24, 128.85, 129.67, 133.81, 138.67, 171.78. Accurate mass: found [*M* + H] = 306.0370; calculated 306.0371.

## Refinement details   

Crystal data, data collection and structure refinement details are summarized in Table 6 ▸. The carbon-bound H atoms were placed in calculated positions (C—H = 0.95 Å) and were included in the refinement in the riding-model approximation, with *U*_iso_(H) set to 1.2–1.5*U*_eq_(C). The N-bound H atoms were refined with a distance restraint of 0.88±0.01 Å, and with *U*_iso_(H) = 1.2*U*_eq_(N). Owing to poor agreement, two reflections, *i.e*. (1 5 11) and (1 7 15), were omitted from the final cycles of refinement.

## Supplementary Material

Crystal structure: contains datablock(s) I, global. DOI: 10.1107/S2056989019003980/hb7809sup1.cif

Structure factors: contains datablock(s) I. DOI: 10.1107/S2056989019003980/hb7809Isup2.hkl

Supporting information file. DOI: 10.1107/S2056989019003980/hb7809Isup3.cml

CCDC reference: 1905136

Additional supporting information:  crystallographic information; 3D view; checkCIF report

## Figures and Tables

**Table 1:** Hydrogen-bond geometry (Å, °)

*D*—H⋯*A*	*D*—H	H⋯*A*	*D*⋯*A*	*D*—H⋯*A*
N2—H2*N*⋯N4	0.88 (4)	1.96 (4)	2.840 (7)	176 (8)
N3—H3*N*⋯O1^i^	0.88 (3)	2.09 (4)	2.930 (7)	160 (5)
N5—H5*N*⋯N1	0.88 (5)	2.04 (4)	2.900 (7)	165 (6)
N6—H6*N*⋯O4^ii^	0.89 (3)	2.07 (3)	2.956 (6)	175 (6)
C4—H4⋯O2^iii^	0.95	2.55	3.450 (8)	159

Symmetry codes: (i) 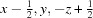; (ii) 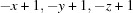; (iii) 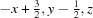.

**Table 2:** Summary of short inter­atomic contacts (Å) in (I)

Contact	Distance	Symmetry operation
H10⋯H16	2.17	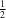 + *x*, 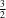 − *y*, 1 − *z*
H11⋯O4	2.54	− 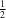 + *x*, 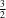 − *y*, 1 − *z*
H19⋯O2	2.51	− 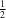 + *x*, *y*, 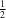 − *z*
C7⋯H3	2.78	− 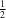 + *x*, *y*, 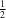 − *z*
C20⋯C23	3.315 (10)	−1 + *x*, *y*, *z*

**Table 3:** Summary of π–π contacts (Å) in (I)

Ring 1	Ring 2	Distance	Symmetry operation
*Cg*(C8–C13)	*Cg*(S3,C14,N4,C20–C15)	3.848 (4)	*x*, *y*, *z*
*Cg*(C8–C13)	*Cg*(C15–C20)	3.891 (5)	*x*, *y*, *z*
*Cg*(S3—C14—N4—C20—C15)	*Cg*(C21–C26)	3.923 (4)	− 1 + *x*, *y*, *z*

**Table 4:** Percentage contributions of inter­atomic contacts to the Hirshfeld surface for (I)

	Percentage contribution		
Contact	S1-mol­ecule	S3-mol­ecule	overall (I)
H⋯H	34.0	31.7	34.5
O⋯H/H⋯O	21.1	21.5	26.2
C⋯H/H⋯C	21.6	16.6	16.5
S⋯H/H⋯S	6.9	11.4	11.9
N⋯H/H⋯N	8.1	8.2	4.1
C⋯C	4.2	7.2	2.8
C⋯O/O⋯C	0.9	0.9	1.1
C⋯S/S⋯C	1.2	1.2	1.6
C⋯N/N⋯C	0.7	1.3	0.4
O⋯O	0.6	0.0	0.4
S⋯N/N⋯S	0.8	0.0	0.5

**Table 5:** Inter­action energies (kJ mol^−1^) for selected close contacts in (I)

contact	*E*_electrostatic_	*E*_polarization_	*E*_dispersion_	*E*_exchange-repulsion_	*E*_total_
N2—H2*N*⋯N4	−121.4	−30.0	−81.2	158.8	−123.1
N5—H5*N*⋯N1	−121.4	−30.0	−81.2	158.8	−123.1
N3—H3*N*⋯O1^i^	−39.3	−10.5	−40.0	52.1	−51.9
N6—H6*N*⋯O4^ii^	−69.0	−15.7	−29.7	65.0	−70.3
C4—H4⋯O2^iii^	−4.2	−1.3	−15.4	13.4	−10.6
C19—H19⋯O2^i^	−1.0	−2.0	−14.8	11.6	−8.2
C11—H11⋯O4^iv^	−10.2	−2.0	−7.6	6.8	−14.7

Symmetry codes: (i) *x* − 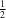, *y*, −*z* + 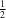; (ii) −*x* + 1, −*y* + 1, −*z* + 1; (iii) −*x* + 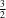, *y* − 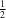, *z*; (iv) *x* − 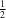, −*y* + 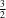, −*z* + 1

**Table 6:** Experimental details

Crystal data	
Chemical formula	C_13_H_11_N_3_O_2_S_2_
*M*_r_	305.37
Crystal system, space group	Orthorhombic, *P* *b* *c* *a*
Temperature (K)	100
*a*, *b*, *c* (Å)	8.9083 (7), 21.6499 (9), 29.4778 (18)
*V* (Å^3^)	5685.2 (6)
*Z*	16
Radiation type	Synchrotron, λ = 0.6889 Å
μ (mm^−1^)	0.34
Crystal size (mm)	0.01 × 0.01 × 0.01

Data collection	
Diffractometer	Three-circle diffractometer
Absorption correction	Empirical (using intensity measurements) (*AIMLESS *CCP4**; Evans, 2006 ▸)
*T*_min_, *T*_max_	0.996, 1.000
No. of measured, independent and observed [*I* > 2σ(*I*)] reflections	15484, 5451, 2289
*R*_int_	0.199
(sin θ/λ)_max_ (Å^−1^)	0.613

Refinement	
*R*[*F* ^2^ > 2σ(*F* ^2^)], *wR*(*F* ^2^), *S*	0.083, 0.246, 0.77
No. of reflections	5451
No. of parameters	373
No. of restraints	4
H-atom treatment	H atoms treated by a mixture of independent and constrained refinement
Δρ_max_, Δρ_min_ (e Å^−3^)	0.56, −0.44

Computer programs: *GDA* (http://www.opengda.org/OpenGDA.html), *XIA2 0.4.0.370-g47f3bc3* (Winter, 2010 ▸), *SHELXS97* (Sheldrick, 2008 ▸), *SHELXL2018* (Sheldrick, 2015 ▸), *ORTEP-3 for Windows* (Farrugia, 2012 ▸), *Q-Mol* Gans & Shalloway (2001 ▸) and *DIAMOND* (Brandenburg, 2006 ▸) and *publCIF* (Westrip, 2010 ▸).

## supplementary crystallographic information

### Crystal data

**Table:**

C_13_H_11_N_3_O_2_S_2_	*D*_x_ = 1.427 Mg m^−^^3^
*M**_r_* = 305.37	Synchrotron radiation, λ = 0.6889 Å
Orthorhombic, *P**b**c**a*	Cell parameters from 2386 reflections
*a* = 8.9083 (7) Å	θ = 2.5–28.6°
*b* = 21.6499 (9) Å	µ = 0.34 mm^−^^1^
*c* = 29.4778 (18) Å	*T* = 100 K
*V* = 5685.2 (6) Å^3^	Cube, colourless
*Z* = 16	0.01 × 0.01 × 0.01 mm
*F*(000) = 2528	

### Data collection

**Table:**

Three-circle diffractometer	5451 independent reflections
Radiation source: synchrotron, DLS beamline I19, undulator	2289 reflections with *I* > 2σ(*I*)
Si 111, double crystal monochromator	*R*_int_ = 0.199
Detector resolution: 5.81 pixels mm^-1^	θ_max_ = 25.0°, θ_min_ = 1.8°
profile data from ω–scans	*h* = −10→10
Absorption correction: empirical (using intensity measurements) (*AIMLESS CCP4*; Evans, 2006)	*k* = −10→26
*T*_min_ = 0.996, *T*_max_ = 1.000	*l* = −31→35
15484 measured reflections	

### Refinement

**Table:**

Refinement on *F*^2^	Primary atom site location: structure-invariant direct methods
Least-squares matrix: full	Secondary atom site location: difference Fourier map
*R*[*F*^2^ > 2σ(*F*^2^)] = 0.083	Hydrogen site location: mixed
*wR*(*F*^2^) = 0.246	H atoms treated by a mixture of independent and constrained refinement
*S* = 0.77	*w* = 1/[σ^2^(*F*_o_^2^) + (0.1*P*)^2^] where *P* = (*F*_o_^2^ + 2*F*_c_^2^)/3
5451 reflections	(Δ/σ)_max_ = 0.001
373 parameters	Δρ_max_ = 0.56 e Å^−^^3^
4 restraints	Δρ_min_ = −0.44 e Å^−^^3^

### Special details

**Table:**

Geometry. All esds (except the esd in the dihedral angle between two l.s. planes) are estimated using the full covariance matrix. The cell esds are taken into account individually in the estimation of esds in distances, angles and torsion angles; correlations between esds in cell parameters are only used when they are defined by crystal symmetry. An approximate (isotropic) treatment of cell esds is used for estimating esds involving l.s. planes.
Refinement. The sample was small and data were measured on the DLS beamline I19 with synchrotron radiation. The crystal dimensions were not recorded and assumed to be 0.01 x 0.01 x 0.01 mm^3^. Data were truncated at θ = 25.0 so data completeness was > 99%. The value of R_int_ is high but, the ordered structure has been determined unambiguously. The GoF is poor but, this probably reflects the limited data available for the sample investigated.

### Fractional atomic coordinates and isotropic or equivalent isotropic displacement parameters (Å^2^)

**Table:**

	*x*	*y*	*z*	*U*_iso_*/*U*_eq_	
S1	0.60554 (19)	0.58255 (6)	0.24704 (5)	0.0503 (4)	
S2	0.51420 (19)	0.75258 (5)	0.27917 (5)	0.0454 (4)	
O1	0.6648 (5)	0.73163 (18)	0.28878 (14)	0.0592 (12)	
O2	0.4882 (6)	0.79329 (17)	0.24171 (13)	0.0646 (13)	
N1	0.5102 (6)	0.55146 (19)	0.32861 (15)	0.0514 (13)	
N2	0.4129 (6)	0.64482 (19)	0.30137 (16)	0.0481 (12)	
H2N	0.353 (6)	0.645 (3)	0.3252 (13)	0.058*	
N3	0.4169 (6)	0.68926 (18)	0.26694 (16)	0.0440 (12)	
H3N	0.329 (3)	0.695 (3)	0.2542 (18)	0.053*	
C1	0.4979 (7)	0.5945 (2)	0.29675 (19)	0.0482 (15)	
C2	0.6739 (7)	0.5141 (2)	0.2705 (2)	0.0513 (15)	
C3	0.7784 (8)	0.4730 (2)	0.2528 (2)	0.0598 (18)	
H3	0.819254	0.478886	0.223372	0.072*	
C4	0.8222 (9)	0.4221 (3)	0.2800 (3)	0.075 (2)	
H4	0.896758	0.394257	0.269346	0.090*	
C5	0.7563 (9)	0.4127 (2)	0.3222 (3)	0.071 (2)	
H5	0.784616	0.377377	0.339315	0.085*	
C6	0.6517 (9)	0.4526 (3)	0.3401 (2)	0.0657 (19)	
H6	0.608183	0.445225	0.369067	0.079*	
C7	0.6109 (7)	0.5049 (2)	0.3141 (2)	0.0548 (17)	
C8	0.4406 (7)	0.7849 (2)	0.32924 (17)	0.0452 (15)	
C9	0.4975 (9)	0.7651 (3)	0.3714 (2)	0.0587 (18)	
H9	0.576259	0.735541	0.372649	0.070*	
C10	0.4387 (13)	0.7887 (4)	0.4107 (2)	0.093 (3)	
H10	0.476103	0.775303	0.439199	0.111*	
C11	0.3247 (15)	0.8321 (5)	0.4089 (4)	0.114 (4)	
H11	0.284576	0.848228	0.436261	0.137*	
C12	0.2685 (11)	0.8522 (3)	0.3677 (4)	0.095 (3)	
H12	0.190565	0.882071	0.366926	0.114*	
C13	0.3267 (8)	0.8284 (3)	0.3273 (2)	0.0628 (18)	
H13	0.288543	0.841850	0.298885	0.075*	
S3	0.2032 (2)	0.65517 (7)	0.46859 (5)	0.0588 (5)	
S4	0.6181 (2)	0.59193 (6)	0.47670 (6)	0.0584 (5)	
O3	0.6133 (6)	0.65715 (17)	0.47139 (16)	0.0706 (14)	
O4	0.6304 (6)	0.56392 (19)	0.52128 (14)	0.0711 (14)	
N4	0.2271 (6)	0.65127 (19)	0.37953 (16)	0.0492 (13)	
N5	0.3982 (7)	0.5865 (2)	0.41732 (17)	0.0651 (16)	
H5N	0.431 (8)	0.569 (3)	0.3921 (13)	0.078*	
N6	0.4514 (7)	0.5658 (2)	0.45953 (17)	0.0601 (15)	
H6N	0.431 (8)	0.5261 (9)	0.464 (2)	0.072*	
C14	0.2839 (8)	0.6284 (2)	0.4175 (2)	0.0534 (16)	
C15	0.0812 (8)	0.7021 (3)	0.4356 (2)	0.0546 (16)	
C16	−0.0275 (8)	0.7435 (2)	0.4501 (2)	0.0553 (16)	
H16	−0.045765	0.749907	0.481528	0.066*	
C17	−0.1100 (8)	0.7757 (3)	0.4169 (2)	0.0606 (18)	
H17	−0.186125	0.803973	0.425736	0.073*	
C18	−0.0795 (9)	0.7660 (3)	0.3712 (2)	0.0596 (18)	
H18	−0.136054	0.787920	0.349092	0.072*	
C19	0.0311 (8)	0.7252 (2)	0.3567 (2)	0.0524 (16)	
H19	0.050144	0.719456	0.325307	0.063*	
C20	0.1132 (7)	0.6931 (2)	0.38891 (19)	0.0479 (15)	
C21	0.7612 (8)	0.5609 (3)	0.4423 (2)	0.0597 (18)	
C22	0.8006 (10)	0.5918 (3)	0.4026 (3)	0.075 (2)	
H22	0.753526	0.629700	0.394779	0.090*	
C23	0.9117 (10)	0.5660 (4)	0.3740 (3)	0.086 (3)	
H23	0.936312	0.585303	0.345993	0.103*	
C24	0.9838 (11)	0.5125 (4)	0.3871 (4)	0.101 (3)	
H24	1.059314	0.495490	0.368104	0.122*	
C25	0.9477 (11)	0.4826 (4)	0.4283 (3)	0.095 (3)	
H25	1.000034	0.446322	0.437183	0.114*	
C26	0.8351 (9)	0.5066 (3)	0.4555 (3)	0.074 (2)	
H26	0.808163	0.486493	0.483000	0.089*	

### Atomic displacement parameters (Å^2^)

**Table:**

	*U*^11^	*U*^22^	*U*^33^	*U*^12^	*U*^13^	*U*^23^
S1	0.0531 (11)	0.0425 (7)	0.0551 (9)	−0.0019 (6)	0.0039 (7)	−0.0034 (6)
S2	0.0498 (11)	0.0427 (7)	0.0438 (8)	−0.0055 (6)	0.0029 (7)	−0.0036 (6)
O1	0.048 (3)	0.061 (2)	0.068 (3)	0.004 (2)	0.000 (2)	−0.020 (2)
O2	0.097 (4)	0.053 (2)	0.044 (2)	−0.019 (2)	0.005 (2)	0.0073 (17)
N1	0.060 (4)	0.042 (2)	0.053 (3)	0.004 (2)	0.001 (2)	−0.004 (2)
N2	0.049 (4)	0.044 (2)	0.051 (3)	0.004 (2)	0.007 (2)	0.009 (2)
N3	0.047 (3)	0.036 (2)	0.049 (3)	0.002 (2)	−0.003 (2)	0.0000 (18)
C1	0.049 (4)	0.038 (3)	0.058 (4)	−0.002 (2)	−0.003 (3)	−0.005 (2)
C2	0.042 (4)	0.041 (3)	0.071 (4)	−0.006 (2)	0.003 (3)	−0.007 (3)
C3	0.061 (5)	0.037 (3)	0.081 (5)	−0.006 (3)	0.005 (4)	−0.011 (3)
C4	0.069 (6)	0.040 (3)	0.118 (7)	0.000 (3)	0.012 (5)	−0.019 (3)
C5	0.076 (6)	0.039 (3)	0.097 (6)	0.009 (3)	−0.006 (4)	−0.001 (3)
C6	0.079 (6)	0.047 (3)	0.071 (4)	0.003 (3)	0.000 (4)	0.001 (3)
C7	0.059 (5)	0.038 (3)	0.067 (4)	−0.001 (3)	−0.007 (3)	−0.007 (3)
C8	0.064 (5)	0.033 (2)	0.039 (3)	−0.009 (3)	0.014 (3)	−0.003 (2)
C9	0.076 (6)	0.053 (3)	0.047 (4)	−0.013 (3)	0.007 (3)	0.000 (3)
C10	0.143 (9)	0.091 (5)	0.045 (4)	−0.047 (6)	0.019 (5)	−0.025 (4)
C11	0.139 (10)	0.105 (7)	0.098 (7)	−0.072 (7)	0.075 (7)	−0.058 (6)
C12	0.080 (7)	0.059 (4)	0.146 (9)	−0.010 (4)	0.042 (6)	−0.043 (5)
C13	0.051 (5)	0.049 (3)	0.088 (5)	0.003 (3)	0.005 (4)	−0.008 (3)
S3	0.0749 (14)	0.0598 (8)	0.0417 (9)	0.0072 (8)	−0.0022 (8)	0.0074 (6)
S4	0.0721 (14)	0.0445 (7)	0.0587 (10)	0.0039 (7)	−0.0046 (9)	−0.0027 (6)
O3	0.085 (4)	0.045 (2)	0.082 (3)	−0.005 (2)	0.013 (3)	0.002 (2)
O4	0.095 (4)	0.060 (2)	0.058 (3)	0.005 (2)	−0.008 (2)	−0.005 (2)
N4	0.058 (4)	0.046 (2)	0.044 (3)	−0.003 (2)	0.001 (2)	0.007 (2)
N5	0.087 (5)	0.070 (3)	0.038 (3)	0.026 (3)	−0.003 (3)	0.009 (2)
N6	0.081 (5)	0.043 (2)	0.056 (3)	−0.001 (3)	−0.003 (3)	0.010 (2)
C14	0.062 (5)	0.045 (3)	0.053 (4)	−0.003 (3)	0.003 (3)	0.002 (3)
C15	0.064 (5)	0.057 (3)	0.042 (3)	−0.007 (3)	−0.007 (3)	0.004 (3)
C16	0.060 (5)	0.058 (3)	0.048 (4)	0.002 (3)	0.001 (3)	0.002 (3)
C17	0.064 (5)	0.056 (3)	0.062 (4)	0.011 (3)	−0.006 (3)	−0.002 (3)
C18	0.075 (6)	0.062 (3)	0.041 (3)	0.016 (3)	−0.005 (3)	−0.001 (3)
C19	0.067 (5)	0.048 (3)	0.042 (3)	−0.006 (3)	0.000 (3)	0.006 (2)
C20	0.046 (4)	0.050 (3)	0.048 (4)	−0.011 (3)	−0.001 (3)	0.008 (2)
C21	0.068 (5)	0.052 (3)	0.060 (4)	−0.007 (3)	−0.003 (3)	−0.003 (3)
C22	0.091 (7)	0.053 (3)	0.082 (5)	−0.019 (4)	0.007 (5)	−0.012 (3)
C23	0.099 (7)	0.071 (5)	0.088 (6)	−0.036 (5)	0.022 (5)	−0.029 (4)
C24	0.068 (7)	0.098 (6)	0.139 (9)	−0.015 (5)	0.024 (6)	−0.053 (6)
C25	0.081 (7)	0.076 (5)	0.127 (8)	0.011 (4)	0.010 (6)	−0.023 (5)
C26	0.070 (6)	0.065 (4)	0.087 (5)	0.015 (4)	0.003 (4)	−0.013 (4)

### Geometric parameters (Å, º)

**Table:**

S1—C2	1.746 (6)	S3—C14	1.766 (6)
S1—C1	1.770 (6)	S3—C15	1.777 (6)
S2—O2	1.432 (4)	S4—O3	1.421 (4)
S2—O1	1.444 (5)	S4—O4	1.451 (5)
S2—N3	1.662 (5)	S4—N6	1.668 (6)
S2—C8	1.760 (5)	S4—C21	1.761 (7)
N1—C1	1.327 (7)	N4—C14	1.325 (7)
N1—C7	1.416 (7)	N4—C20	1.388 (8)
N2—C1	1.334 (7)	N5—C14	1.365 (8)
N2—N3	1.399 (6)	N5—N6	1.405 (7)
N2—H2N	0.880 (10)	N5—H5N	0.884 (10)
N3—H3N	0.877 (10)	N6—H6N	0.886 (10)
C2—C3	1.389 (9)	C15—C16	1.387 (9)
C2—C7	1.415 (9)	C15—C20	1.419 (8)
C3—C4	1.418 (9)	C16—C17	1.409 (9)
C3—H3	0.9500	C16—H16	0.9500
C4—C5	1.390 (10)	C17—C18	1.392 (9)
C4—H4	0.9500	C17—H17	0.9500
C5—C6	1.376 (10)	C18—C19	1.389 (9)
C5—H5	0.9500	C18—H18	0.9500
C6—C7	1.416 (8)	C19—C20	1.384 (8)
C6—H6	0.9500	C19—H19	0.9500
C8—C13	1.386 (9)	C21—C22	1.394 (9)
C8—C9	1.410 (8)	C21—C26	1.402 (9)
C9—C10	1.369 (10)	C22—C23	1.415 (11)
C9—H9	0.9500	C22—H22	0.9500
C10—C11	1.383 (15)	C23—C24	1.380 (12)
C10—H10	0.9500	C23—H23	0.9500
C11—C12	1.382 (14)	C24—C25	1.413 (13)
C11—H11	0.9500	C24—H24	0.9500
C12—C13	1.397 (11)	C25—C26	1.387 (11)
C12—H12	0.9500	C25—H25	0.9500
C13—H13	0.9500	C26—H26	0.9500
			
C2—S1—C1	89.1 (3)	C14—S3—C15	88.3 (3)
O2—S2—O1	119.7 (3)	O3—S4—O4	121.1 (3)
O2—S2—N3	104.8 (2)	O3—S4—N6	106.1 (3)
O1—S2—N3	105.5 (2)	O4—S4—N6	101.5 (3)
O2—S2—C8	110.0 (3)	O3—S4—C21	109.7 (3)
O1—S2—C8	107.8 (3)	O4—S4—C21	107.9 (3)
N3—S2—C8	108.4 (3)	N6—S4—C21	109.9 (3)
C1—N1—C7	109.7 (5)	C14—N4—C20	110.8 (5)
C1—N2—N3	118.2 (5)	C14—N5—N6	117.4 (5)
C1—N2—H2N	116 (4)	C14—N5—H5N	122 (5)
N3—N2—H2N	126 (4)	N6—N5—H5N	120 (5)
N2—N3—S2	115.0 (4)	N5—N6—S4	117.4 (4)
N2—N3—H3N	112 (4)	N5—N6—H6N	111 (4)
S2—N3—H3N	117 (4)	S4—N6—H6N	118 (5)
N1—C1—N2	123.2 (5)	N4—C14—N5	122.0 (5)
N1—C1—S1	116.0 (4)	N4—C14—S3	116.3 (5)
N2—C1—S1	120.7 (4)	N5—C14—S3	121.7 (4)
C3—C2—C7	121.1 (5)	C16—C15—C20	121.9 (6)
C3—C2—S1	128.9 (5)	C16—C15—S3	128.9 (5)
C7—C2—S1	109.9 (4)	C20—C15—S3	109.3 (5)
C2—C3—C4	118.0 (6)	C15—C16—C17	118.0 (6)
C2—C3—H3	121.0	C15—C16—H16	121.0
C4—C3—H3	121.0	C17—C16—H16	121.0
C5—C4—C3	120.2 (6)	C18—C17—C16	119.8 (6)
C5—C4—H4	119.9	C18—C17—H17	120.1
C3—C4—H4	119.9	C16—C17—H17	120.1
C6—C5—C4	122.6 (6)	C19—C18—C17	122.1 (6)
C6—C5—H5	118.7	C19—C18—H18	119.0
C4—C5—H5	118.7	C17—C18—H18	119.0
C5—C6—C7	117.8 (6)	C20—C19—C18	118.9 (6)
C5—C6—H6	121.1	C20—C19—H19	120.5
C7—C6—H6	121.1	C18—C19—H19	120.5
N1—C7—C6	124.6 (6)	C19—C20—N4	125.2 (5)
N1—C7—C2	115.2 (5)	C19—C20—C15	119.3 (6)
C6—C7—C2	120.2 (6)	N4—C20—C15	115.4 (5)
C13—C8—C9	120.4 (6)	C22—C21—C26	121.2 (7)
C13—C8—S2	120.6 (5)	C22—C21—S4	118.9 (5)
C9—C8—S2	119.0 (5)	C26—C21—S4	119.9 (5)
C10—C9—C8	119.6 (8)	C21—C22—C23	119.2 (7)
C10—C9—H9	120.2	C21—C22—H22	120.4
C8—C9—H9	120.2	C23—C22—H22	120.4
C9—C10—C11	120.1 (8)	C24—C23—C22	119.3 (8)
C9—C10—H10	119.9	C24—C23—H23	120.4
C11—C10—H10	119.9	C22—C23—H23	120.4
C12—C11—C10	120.9 (7)	C23—C24—C25	121.4 (8)
C12—C11—H11	119.6	C23—C24—H24	119.3
C10—C11—H11	119.6	C25—C24—H24	119.3
C11—C12—C13	119.9 (8)	C26—C25—C24	119.3 (8)
C11—C12—H12	120.1	C26—C25—H25	120.3
C13—C12—H12	120.1	C24—C25—H25	120.3
C8—C13—C12	119.1 (7)	C25—C26—C21	119.5 (8)
C8—C13—H13	120.4	C25—C26—H26	120.2
C12—C13—H13	120.4	C21—C26—H26	120.2
			
C1—N2—N3—S2	−104.5 (5)	C14—N5—N6—S4	−106.6 (6)
O2—S2—N3—N2	−173.6 (4)	O3—S4—N6—N5	49.7 (5)
O1—S2—N3—N2	59.1 (4)	O4—S4—N6—N5	177.1 (4)
C8—S2—N3—N2	−56.2 (5)	C21—S4—N6—N5	−68.8 (5)
C7—N1—C1—N2	−178.6 (5)	C20—N4—C14—N5	−179.6 (6)
C7—N1—C1—S1	0.1 (6)	C20—N4—C14—S3	0.5 (7)
N3—N2—C1—N1	175.5 (5)	N6—N5—C14—N4	179.2 (5)
N3—N2—C1—S1	−3.1 (7)	N6—N5—C14—S3	−0.9 (8)
C2—S1—C1—N1	−0.1 (5)	C15—S3—C14—N4	−0.3 (5)
C2—S1—C1—N2	178.6 (5)	C15—S3—C14—N5	179.9 (6)
C1—S1—C2—C3	−177.5 (6)	C14—S3—C15—C16	−178.1 (6)
C1—S1—C2—C7	0.1 (5)	C14—S3—C15—C20	−0.1 (4)
C7—C2—C3—C4	−1.1 (9)	C20—C15—C16—C17	1.9 (9)
S1—C2—C3—C4	176.3 (5)	S3—C15—C16—C17	179.7 (5)
C2—C3—C4—C5	2.8 (10)	C15—C16—C17—C18	−0.9 (10)
C3—C4—C5—C6	−2.4 (11)	C16—C17—C18—C19	−0.2 (10)
C4—C5—C6—C7	0.0 (11)	C17—C18—C19—C20	0.2 (10)
C1—N1—C7—C6	178.9 (6)	C18—C19—C20—N4	−179.7 (6)
C1—N1—C7—C2	0.0 (7)	C18—C19—C20—C15	0.8 (9)
C5—C6—C7—N1	−177.1 (6)	C14—N4—C20—C19	179.9 (6)
C5—C6—C7—C2	1.7 (10)	C14—N4—C20—C15	−0.6 (7)
C3—C2—C7—N1	177.7 (6)	C16—C15—C20—C19	−1.8 (9)
S1—C2—C7—N1	−0.1 (7)	S3—C15—C20—C19	180.0 (5)
C3—C2—C7—C6	−1.2 (9)	C16—C15—C20—N4	178.6 (6)
S1—C2—C7—C6	−179.0 (5)	S3—C15—C20—N4	0.4 (6)
O2—S2—C8—C13	23.5 (6)	O3—S4—C21—C22	−28.5 (7)
O1—S2—C8—C13	155.6 (5)	O4—S4—C21—C22	−162.4 (5)
N3—S2—C8—C13	−90.6 (5)	N6—S4—C21—C22	87.8 (6)
O2—S2—C8—C9	−157.6 (5)	O3—S4—C21—C26	150.3 (6)
O1—S2—C8—C9	−25.5 (5)	O4—S4—C21—C26	16.5 (7)
N3—S2—C8—C9	88.3 (5)	N6—S4—C21—C26	−93.4 (6)
C13—C8—C9—C10	0.5 (9)	C26—C21—C22—C23	3.4 (11)
S2—C8—C9—C10	−178.3 (5)	S4—C21—C22—C23	−177.8 (6)
C8—C9—C10—C11	−0.4 (11)	C21—C22—C23—C24	−3.2 (11)
C9—C10—C11—C12	0.1 (13)	C22—C23—C24—C25	0.8 (12)
C10—C11—C12—C13	0.3 (13)	C23—C24—C25—C26	1.5 (13)
C9—C8—C13—C12	−0.2 (9)	C24—C25—C26—C21	−1.3 (13)
S2—C8—C13—C12	178.6 (5)	C22—C21—C26—C25	−1.1 (11)
C11—C12—C13—C8	−0.2 (11)	S4—C21—C26—C25	−179.9 (6)

### Hydrogen-bond geometry (Å, º)

**Table:**

*D*—H···*A*	*D*—H	H···*A*	*D*···*A*	*D*—H···*A*
N2—H2*N*···N4	0.88 (4)	1.96 (4)	2.840 (7)	176 (8)
N3—H3*N*···O1^i^	0.88 (3)	2.09 (4)	2.930 (7)	160 (5)
N5—H5*N*···N1	0.88 (5)	2.04 (4)	2.900 (7)	165 (6)
N6—H6*N*···O4^ii^	0.89 (3)	2.07 (3)	2.956 (6)	175 (6)
C4—H4···O2^iii^	0.95	2.55	3.450 (8)	159

Symmetry codes: (i) *x*−1/2, *y*, −*z*+1/2; (ii) −*x*+1, −*y*+1, −*z*+1; (iii) −*x*+3/2, *y*−1/2, *z*.
